# Unravelling Teacher Job Satisfaction: The Contribution of Collective Efficacy and Emotions Towards Professional Role

**DOI:** 10.3390/ijerph17030736

**Published:** 2020-01-23

**Authors:** Ilaria Buonomo, Caterina Fiorilli, Paula Benevene

**Affiliations:** Department of Human Sciences, LUMSA University, 00193 Rome, Italy; fiorilli@lumsa.it (C.F.); benevene@lumsa.it (P.B.)

**Keywords:** job satisfaction, emotions, efficacy beliefs, schools, teachers

## Abstract

*Purpose*: The purpose of this paper is to explore whether, and to what extent, collective beliefs and emotions towards professional role could predict job satisfaction, above and beyond the role of self-efficacy and emotions towards students. More specifically, we expected job satisfaction to be incrementally predicted by beliefs and emotions related to professional role (collective efficacy and role-related hedonic balance). *Design/Methodology/Approach*: The analysis was performed through the administration of a questionnaire to 266 Italian secondary school teachers. After having assessed measures of reliability, correlational analyses and a hierarchical regression model were performed. *Findings*: Results showed that collective efficacy and hedonic balance related to professional role have a unique effect on job satisfaction, accounting for nearly the 30% of its variance. *Research Limitations/Implications*: Despite some limitations related to the cross-sectional design, the study suggests a practical implication for teacher training, as well as underlying the need to study schools from an organizational point of view. *Originality/value*: The paper contributes to the psychological research on the role of the organizational dimensions in teachers’ well-being at work.

## 1. Introduction

Recent international surveys have focused on the increasing challenges that teachers are required to face during their daily job (e.g., multicultural classes, students with special educational needs or antisocial behaviors, new technologies to be implemented, etc.) [1]. Current psychological literature widely addresses the role of teachers’ personal resources [2,3,4,5,6,7], relationships with students [8,9], and individual professional resources [10,11] in managing the mentioned challenges. Despite the meaningful role of the teacher’s individual contributions to these issues, collaborative practices and strategies seem to better fit such needs [12,13]. In light of these socio-cultural changes, it seems important for schools to improve and reinforce their organizational dimension by constructing a stronger organizational identity. According to Schein, organizational changes require the perceptual, emotional, and cognitive involvement of each worker, so that, by valuing worker beliefs and emotions when planning and implementing changes, the adjustments can be pursued by the whole organization [14]. For this reason, it seems helpful to consider teacher beliefs and emotions towards their individual and collective job experience. It is likely that by better understanding how teachers perceive the collective dimensions of teaching, researchers and practitioners could provide better instructions to principals and policy makers to improve school effectiveness and well-being. With this regard, teachers’ job satisfaction may be an effective starting point when it comes to strengthening teachers’ involvement in the organizational facet of school. Studies regarding non-teaching professionals indeed showed that job satisfaction is related to workers’ attitudes towards organizational change and readiness to change [15,16]. Building on these considerations, this work aims to define whether and how cognitive and affective aspects regarding collective dimensions of teaching (i.e., collective efficacy and emotions related to the professional role) may have a role in teacher job satisfaction, above and beyond individual dimensions (i.e., self-efficacy, incremental beliefs, and emotions towards students).

### 1.1. Teachers’ Job Satisfaction

Job satisfaction refers to a sense of fulfillment, gratification, and satisfaction resulting from engaging in an occupation [17]. More specifically, it refers to the degree to which a person feels that his or her job-related needs are being met [18,19]. Malinen and Savolainen recently classified the variables influencing job satisfaction in teachers using three dimensions: organizational aspects (work conditions, relationships, perceived autonomy and support) [20,21,22], cognitive factors (efficacy beliefs) [23,24,25], and affective factors (stress, burnout) [24,25]. Before this classification, Butt and colleagues [26] showed that teachers are generally more satisfied with the cognitive and affective dimensions of work regarding their personal experience of teaching (e.g., perception of the work itself, professional growth, self-efficacy) than with cognitive and affective aspects regarding collective or organizational dimensions (e.g., working conditions and workload, salary, social representation of the profession, relationships with colleagues, leadership styles). Available literature on the relationship between teacher job satisfaction and efficacy beliefs, incremental beliefs, and emotions, in both their individual and collective facets, will be described in the next sections.

### 1.2. Teachers’ Efficacy Beliefs and Their Effect on Job Satisfaction

Teachers’ self-efficacy is “the teacher’s belief in his or her capability to organize and execute the courses of action required to successfully accomplish a specific teaching task in a particular context” ([27], p. 232). When teachers feel confident in their professional abilities, they feel more control over their daily tasks [27,28], higher motivation [29], and positive expectations towards their job [30], which, in turn, lead to actual better results [31]. Consistently, confident teachers tend to adapt their strategies, practices, and teaching style according to the specific needs of their students in each classroom [28,32,33,34].

Nevertheless, each teacher is part of a job community, as well as part of a broader organization. Feeling confident in one’s own abilities is indeed not sufficient when considering the effects of efficacy beliefs on actual teaching outcomes [28,35]. Accordingly, teachers are influenced by educational policies [36,37], principals [38], colleagues, students, and families [38,39]. Moreover, all these aspects relate to one another [40]. Consistently, it seems useful to take into account collective efficacy beliefs [41]. Hoy, Davis and Pape [42] described school collective efficacy as “the perceptions of teachers in a school that the faculty as a whole can organize and execute the courses of action required to have a positive effect on students” (p. 728). Similar to self-efficacy, collective beliefs are related to positive experiences at school (e.g., past successes, support from colleagues and principals) [43], as well as social and educational changes and policies [44,45]. Teaching communities with high efficacy are beneficial for teachers in terms of high motivation and resilience [25,30], and for students in terms of academic achievement and motivation [46,47]. Several studies reported that efficacy beliefs contribute significantly to job satisfaction [23,25,28,48]. This has been shown for self-efficacy beliefs [23,28], but is still not defined for collective efficacy. While some authors showed that collective efficacy related to student discipline, instructional strategies, and adequacy of school resources is positively associated to job satisfaction, others [25,48,49,50] failed to show a direct effect on job satisfaction. Despite these gaps, current literature suggests that beliefs about personal successful teaching experiences and working in a school with good policies may heighten teachers’ job satisfaction.

### 1.3. Teachers’ Incremental Beliefs and Their Effect on Job Satisfaction

Carol Dweck defines incremental beliefs as the belief that one’s own abilities and competencies may improve by means of experience and learning [51]. These beliefs are opposed to entity beliefs, according to which people believe their abilities are fixed and cannot be modified. When people believe they will become more and more capable of doing something, they pursue goals related to personal growth; on the contrary, when people feel their abilities are not modifiable, they pursue goals related to show others what they can do [51,52]. While incremental beliefs may act as a source of satisfaction as they meet the need to become more capable in a specific context, entity beliefs may contribute to lower satisfaction levels as people may feel they can not properly manage their tasks. To the best of our knowledge, no studies addressed the role of teachers’ incremental beliefs towards their own teaching experience. At the same time, research with adult students showed that incremental beliefs are associated with academic success, positive emotions toward the learning experience, and higher motivation and self-esteem [53,54]. Such studies suggest that teachers may show a similar pattern of association between incremental beliefs and their teaching experience. Namely, it is possible that when teachers develop positive, incremental attitudes towards their professional growth, they are more likely to work better, experience positive emotions, and feel more capable as professionals. This, in turn, could lead to higher job satisfaction.

### 1.4. Teachers’ Emotions and Hedonic Balance

Emotions and emotional regulation are at the core of the teaching profession [8,41,55,56,57]. Consistently, when teachers perceive themselves as able to properly manage their emotions, they perform and feel better about their job [8,58,59]. Teachers with good levels of emotional regulation better manage relationships (with students, colleagues, and principals), as well as daily stressors [60,61,62,63]. Diener and colleagues [64,65] claimed the regulation of positive and negative emotions could be synthesized in a construct called hedonic balance. This construct refers to the balance of positive and negative emotions, measured as the difference between the two. Some studies addressed the association between hedonic balance and other well-being-related constructs, such as life satisfaction [66]. Furthermore, hedonic balance may account for job-related well-being and job satisfaction. Studies about the relationship between positive emotions and job satisfaction indeed suggest that hedonic balance could be associated with job satisfaction [24,67,68]. Consistently, Boekaerts claimed that job satisfaction is associated with behavioral and affective self-regulation [69]. To the best of our knowledge, while several studies (some of these already mentioned in this introduction) have addressed emotions and hedonic balance related to the personal experiences of teachers at work, few studies have considered their organizational facets (e.g., hedonic balance related to the professional role) [59]. Among these, no studies have considered these organizational dimensions as related to job satisfaction in teachers.

### 1.5. The Present Study

Current research informs about the impact of the mentioned variables (i.e., efficacy beliefs, incremental beliefs, emotions) on teaching, but, to the best of our knowledge, does not provide a model that simultaneously accounts for the effects of these dimensions on teachers’ job satisfaction, differentiating among their personal and organizational facets. Building on current literature, it seems that collective efficacy beliefs and emotions towards professional role, if compared with self-efficacy, incremental beliefs, and emotions towards students, may be less related to daily work and become salient when specific events arise (e.g., educational reforms, changes in school organization, need to address specific problems with students, etc.) [70]. For these reasons, we chose to explore whether, and to what extent, collective beliefs and emotions towards the professional role could predict job satisfaction, above and beyond the effect of self-efficacy, incremental beliefs, and emotions towards students.

### 1.6. We Set Two Hypotheses:

**Hypothesis** **1**. *We expected that job satisfaction was positively correlated with efficacy beliefs (i.e., self- and collective efficacy), incremental beliefs, and hedonic balance. Moreover, socio-demographic variables were considered.*

**Hypothesis** **2**. *We expected that job satisfaction was incrementally predicted by beliefs and emotions related to personal experience (self-efficacy, incremental beliefs, and student-related hedonic balance) and beliefs and emotions related to the professional role (collective efficacy and role-related hedonic balance).*

## 2. Methods

### 2.1. Participants

Two hundred and sixty-six secondary school teachers (F = 69.1%) from Center and South Italy were involved. Teachers were aged 26 to 65 (M = 48.95 years; SD = 8.31) and had 1 to 41 years of job experience (M = 21.72; SD = 10.36). They were mainly class (87.1%) and permanent (83.1%) teachers. Participants were recruited at school after principals’ agreement to the study. Consequently, data were gathered face-to-face during collective meetings with teachers in each participant school. Teachers were instructed by informed consent that they could leave the study at any time, and they could ask the researchers for further information either by personal contact or using a specific online module. Data were gathered from September 2015 to March 2016.

### 2.2. Instruments

The Metacognitive Questionnaires for Teachers [45] was administered to measure the following variables:

*Job satisfaction*, measured by 5 items: teachers were asked to define whether they agreed with each item on a 7-point Likert scale (1 = Strongly disagree, 7 = Strongly agree). Cronbach’s Alpha was = 0.835, and McDonalds’ Omega was = 855.

*Incremental beliefs*, measured by a 16-item scale: teachers were asked to define whether they thought they could improve specific teaching abilities thanks to experience or training opportunities using a 9-point Likert scale (1 = not improvable, 9 = totally improvable). Cronbach’s Alpha was = 0.975, and McDonalds’ Omega was = 982.

*Self-efficacy*, measured by 24 items: teachers were asked to evaluate whether they felt effective when approaching daily teaching tasks on a 10-point Likert scale (1 = not effective, 10 = totally effective). Cronbach’s Alpha was = 0.966, and McDonalds’ Omega was = 967.

*Emotions related to relationships with students and to the professional role*, measured by 30 items: teachers were asked to define how frequently they perceived certain emotions by using a 5-point Likert scale (1 = Almost never, 5 = Almost always). Starting from these scales, two further scales were measured: Hedonic balance related to Students (HB-Student) and Hedonic balance related to Professional role (HB-Professional Role), both measured as the difference between positive and negative emotions. Cronbach’s Alpha was = 0.914 for positive student-related emotions, 0.911 for negative student-related emotions, 0.931 for positive profession-related emotions, and 0.916 for negative profession-related emotions. McDonalds’ Omega was = 0.918 for positive student-related emotions, 0.910 for negative student-related emotions, 0.934 for positive profession-related emotions, and 0.915 for negative profession-related emotions.

*Collective efficacy*, measured by a 9-item scale: teachers were asked whether they feel their school is effective in managing daily educational tasks on a 7-point Likert scale (1 = Totally disagree, 7 = Totally agree). Cronbach’s Alpha was = 0.954, and McDonalds’ Omega was = 955.

### 2.3. Analyses Plan

Correlational patterns were calculated to verify whether job satisfaction was associated with social-demographic variables, efficacy, and incremental beliefs and emotions. At this stage, gender was recoded as a dummy variable (1 = male, 0 = female). As sociodemographic variables did not correlate with other variables, these were not included in the further analyses. A hierarchical regression model was conducted to verify whether job satisfaction was predicted by two different blocks of variables: (a) beliefs and emotions related to teachers’ personal experience of school (self-efficacy, incremental beliefs, hedonic balance related to students); (b) beliefs and emotions related to teachers’ professional role (collective efficacy, hedonic balance related to professional role). At each step, the regression equation was assessed for statistically significant variations in the coefficient of determination (R^2^) along with variations in the coefficient of determination; all regression equations and (potential) determinants of job satisfaction were evaluated on the basis of beta weights and their statistical significance. Predicted Probability plots, residuals scatterplots, and Variance Inflation Factor (VIF) <5 and Tolerance >0.80 criteria were used to test, respectively, the normality, homoscedasticity, and non-multicollinearity assumption for regression. All the variables for all the regression models fulfilled the assumptions. Finally, a *p* > 0.001 Mahalanobis distance criterion was used to identify and skip multivariate outliers. Analyses were run with the PROCESS macro [71] for IBM SPSS (vr. 23) and with the psych package in R (vr. 3.5.3) [72].

### 2.4. Data Analyses and Results

Table 1 shows the mean scores, standard deviation values, and patterns of correlation for job satisfaction, efficacy beliefs (self- and collective-efficacy), incremental beliefs, and hedonic balance (student- and role-related). All the associations are significant (*p*< 0.01). The most significant correlations were found among job satisfaction and beliefs and emotions related to professional conditions and role, namely collective efficacy and hedonic balance related to professional role. Overall, the higher the perceived efficacy as a teacher and as a teaching community, and the more incremental beliefs and the higher hedonic balance related to both experience with students and professional role, the higher the job satisfaction.

Hierarchical multiple regression analysis was conducted. Table 2 indicates that the model containing all predictors accounted for 27.8% of variance in job satisfaction. The predictor blocks individually accounted for the following percentages of variance: beliefs and emotions related to teacher’s personal experience of school (self-efficacy, incremental beliefs, hedonic balance related to students) explained 5.3% and beliefs and emotions related to teachers’ professional role (collective efficacy, hedonic balance related to professional role) explained 22.5%. At Step 1 in the regression, beliefs and emotions related to teachers’ personal experience of school showed a significant influence on job satisfaction (F_(3, 274)_ = 5.113, *p* = 0.002). At Step 2, with the introduction of beliefs and emotions related to teachers’ professional role, the explained variance significantly increased, suggesting that collective efficacy and hedonic balance related to professional role significantly predict job satisfaction (F_(5, 272)_ = 20.964, *p* = 0.000). More specifically, both collective efficacy (*p* = 0.000) and hedonic balance related to the professional role (*p* = 0.015) were found to be significant predictors of job satisfaction. Moreover, when introducing the second block, hedonic balance related to students showed a slightly significant effect (*p*= 0.044) on job satisfaction.

## 3. Discussion

Results showed that collective efficacy and hedonic balance related to the professional role have a unique effect on job satisfaction, accounting for nearly the 30% of its variance. Moreover, results showed that hedonic balance related to relationship with students has a significant effect on job satisfaction, but only when taking into account collective efficacy and hedonic balance related to the professional role. The implication on teachers’ job satisfaction will be discussed in the following sections.

### 3.1. The Effect of Collective Efficacy Beliefs on Teachers’ Job Satisfaction

Studies about the role of teachers’ personal beliefs on their job satisfaction [23,25,28,48,73] suggest that teachers are more satisfied when they feel accomplished during their daily job at school. Although personal efficacy beliefs have a main role in influencing professionals’ motivation [29,74] and well-being [75], teaching is not a solitary job. Each teacher is part of a professional community, relates with colleagues and principals and with students and families, must respond to governmental requirements, manage their workload and their personal/organizational resources in order to do an effective job and feel satisfied.

Some studies reported that the perception that one’s own school can manage and respond to daily tasks and specific educational difficulties influences teachers’ job satisfaction, even when considering teachers from different cultures [25]. Our findings confirmed this effect with Italian teachers working in secondary schools by showing that collective efficacy affected job satisfaction, while self-efficacy did not show this effect. With regard to the predictive role of collective efficacy, previous studies have explained the role of collective efficacy on Italian teachers’ job satisfaction by considering collective efficacy as the combined form of each teachers’ self-efficacy beliefs [23]. Moreover, the relative authors suggested that teachers might feel higher job satisfaction when they consider their job as integrated with their colleagues’ job. The explanation may be more complex than this, above all when considering the current working conditions of Italian teachers. Nowadays, the Italian education system may play a significant role in building collective efficacy. Indeed, according to Italian school reform, principals are required to act as managers, leading schools partially autonomous from the State system, in which teachers and principals are called to collaborate in order to compensate for spare public resources. Finally, the Italian education system comprehends heterogeneous teaching roles and contract conditions. With regard to teaching roles, teachers could be regular, special education, or enrichment teachers (namely, teachers who are hired by a principal to enhance and upgrade school curriculum); each teaching role regards different practices and hours of work. At the same time, they could have a permanent or a temporary contract (that could last a couple of months or a school year). This huge complexity may help with the understanding our results, hypothesizing that efficacy beliefs about one’s own teaching community may be more salient than individual efficacy in the classroom when addressing job satisfaction.

Furthermore, it could be hypothesized that the period in which data were gathered could have a role in the effect shown by collective efficacy. Social-political context may directly and indirectly affect satisfaction about one’s own profession and efficacy beliefs [76,77,78] and includes daily relations and interactions, political and historical processes, organizations and institutions, and the interplay among all of them [78]. Starting from this point, it seems useful to consider that this study started right after the implementation of an educational reform which created debates and oppositions among Italian teachers because of several aspects that may have heightened the salience of group- and professional-related beliefs and representations. Among the most debated points of this reform, for example, is that teachers are being evaluated from the principals in terms of successfulness and professional adequacy, and this has been a crucial point in teachers’ reaction to the reform. Considering that job satisfaction is related to the pleasantness to cover one’s own professional role [17] and that collective efficacy is strictly connected to educational organizational dynamics [78], it is possible that participant teachers felt more satisfied according to the level of mastery perceived by the teaching community they belonged to. According to some authors [32,79,80], teachers’ collective efficacy is linked to their sense of community. Overall, this would confirm the idea that collective efficacy regards not only the extent to which teachers believe the school as an organization will have a positive effect on the students [46], but even the ability of the organization to respond to the needs of all its members, teachers included.

### 3.2. The Effect of Hedonic Balance on Teachers’ Job Satisfaction

This study accounted for the hedonic balance as an influential factor on teachers’ job satisfaction. As a dimension of subjective well-being [23], hedonic balance seems to have a direct, positive effect on job satisfaction. Namely, when teachers are able to maximize their perceived positive emotions and minimize negative ones, they feel more satisfied. Interestingly, results showed that hedonic balance related to professional role directly explained job satisfaction, while hedonic balance regarding the relationship with students explained job satisfaction only when hedonic balance related to professional role was taken into account in the regression model. This suggests that attributing positive emotions to one’s own professional role has a stronger impact on job satisfaction than the emotional experience towards students. Moreover, emotions perceived in the classroom impact job satisfaction only when emotions towards the role were taken into account, suggesting that considering one’s own role at work as pleasurable is a condition required to experience positive emotions during the daily teaching practice. This point is consistent with the effect of collective efficacy on job satisfaction in our study and with the TALIS 2013 report [81] in which Italian teachers were reported as feeling less socially recognized and appreciated as professionals than most of their colleagues in Europe.

### 3.3. Limitations

First of all, our research relies on self-report scales, which inform us only about each teacher’s representations. Considering that literature about incremental beliefs suggests that this variable may have a role in predicting job satisfaction, it is possible that the non-significant effect of incrementality beliefs is due to the use of a single-measure approach. Alternatively, the implementation of multi-method studies could help to better understand these issues by taking into account schools’ micro-organization and relationships within the teaching community, as well as descriptions and representations spontaneously vehiculated by the participants. Moreover, a longitudinal design would better consider the implications of educational reform on efficacy beliefs, emotions, and job satisfaction. Finally, a bigger sample that would have allowed a stratification based on socio-demographic variables would have been more informative.

## 4. Conclusions

The role of collective efficacy beliefs on teachers’ job satisfaction found in our study confirm previous studies on other cultural contexts addressing this association. At the same time, the Italian educational system and its heterogeneity and complexity may have a role in explaining the role of collective belief on individual job satisfaction. Similarly, the peculiar condition of Italian teachers, as shown by OCSE TALIS reports [81], may explain the role of hedonic balance towards the professional role. At the same time, results (as well as studies on different professionals [82,83]) suggest that trainings on teamwork at school could improve teacher job satisfaction, as well as positively influence their perception of school community and the profession in general. For example, valuable strategies could include non-school-related activities such as trainings on communication and team-building exercises, as well as workshops aimed at strengthening teachers’ decision making and problem solving at school, such as sessions on educational case scenarios, video-based discussions, and facilitated reflections. It is likely that by strengthening the relational and professional efficacy and effectiveness as professional teams, teachers could better tackle daily challenges, as well as better organize their work, deal with demands, and capitalize on resources.

## Figures and Tables

**Table 1:** Mean, standard deviations, and correlations among variables.

	M	SD	Job Satisfaction	Self-Efficacy	Incremental Beliefs	HB-Students	Collective Efficacy	HB-Professional Role
Job Satisfaction	4.294	1.197	_					
Self-Efficacy	7.250	0.985	0.161 **	_				
Incremental beliefs	6.820	1.638	0.158 **	0.201 **	_			
HB-Students	1.877	0.984	0.187 **	0.460 **	0.271 **	_		
Collective Efficacy	39.032	12.673	0.505 **	0.193 **	0.197 **	0.345 **	_	
HB-Professional Role	1.560	1.153	0.325 **	0.362 **	0.219 **	0.764 **	0.477 **	_

Note.**. *p*< 0.01. HB = Hedonic balance.

**Table 2:** Hierarchical regression model: Effect of beliefs and emotions related to teachers’ personal experience of school and professional role on job satisfaction.

Variables	ΔR^2^	ΔF	β	t	β	t
Step 1	0.053	5.113 ***				
Self-efficacy			0.084	1.268	0.067	1.151
Incremental beliefs			0.109	1.783	0.059	1.088
HB-Students			0.119	1.753	−0.171 *	−2.024
Step 2	0.225	42.422 ***				
Collective Efficacy					0.440 ***	7.450
HB-Professional Role					0.209 *	2.448
R^2^			0.053		0.278	

*Note.* HB=Hedonic balance; ΔR^2^ = change in R2; ΔF = change in F; β = regression coefficient; t = value of t-test statistic; * = *p* < 0.05. *** = *p* < 0.001.
